# Metabolic Alterations Identified in Urine, Plasma and Aortic Smooth Muscle Cells Reflect Cardiovascular Risk in Patients with Programmed Coronary Artery Bypass Grafting

**DOI:** 10.3390/antiox10091369

**Published:** 2021-08-27

**Authors:** Aranzazu Santiago-Hernandez, Paula J. Martinez, Marta Agudiez, Angeles Heredero, Laura Gonzalez-Calero, Alma Yuste-Montalvo, Vanesa Esteban, Gonzalo Aldamiz-Echevarria, Marta Martin-Lorenzo, Gloria Alvarez-Llamas

**Affiliations:** 1 Immunoallergy and Proteomics Laboratory, Department of Immunology, IIS-Fundacion Jimenez Diaz, UAM, 28040 Madrid, Spain; aranzazu_sant@hotmail.com (A.S.-H.); paula_jmg@hotmail.com (P.J.M.); magudiezperez18@gmail.com (M.A.); lauragon.k@gmail.com (L.G.-C.); marta.martin@fjd.es (M.M.-L.); 2 Department of Cardiac Surgery, Fundacion Jimenez Diaz, UAM, 28040 Madrid, Spain; angeles.heredero@quironsalud.es (A.H.); galdamiz@quironsalud.es (G.A.-E.); 3 Allergy and Inmunology Department, IIS-Fundacion Jimenez Diaz, UAM, 28040 Madrid, Spain; almamonyu@gmail.com (A.Y.-M.); vesteban@fjd.es (V.E.); 4 Red de Asma, Reacciones Adversas y Alergicas, Instituto de Salud Carlos III, 28040 Madrid, Spain; 5 Faculty of Medicine and Biomedicine, Alfonso X El Sabio University, 28691 Madrid, Spain; 6 Red de Investigacion Renal (REDINREN), Instituto de Salud Carlos III, 28040 Madrid, Spain

**Keywords:** cardiovascular risk, atherosclerosis, chronic kidney disease, vascular smooth muscle cells, oxidative stress, metabolites, metabolomics, biomarkers

## Abstract

Atherosclerosis is the predominant pathology associated to premature deaths due to cardiovascular disease. However, early intervention based on a personalized diagnosis of cardiovascular risk is very limited. We have previously identified metabolic alterations during atherosclerosis development in a rabbit model and in subjects suffering from an acute coronary syndrome. Here we aim to identify specific metabolic signatures which may set the basis for novel tools aiding cardiovascular risk diagnosis in clinical practice. In a cohort of subjects with programmed coronary artery bypass grafting (CABG), we have performed liquid chromatography and targeted mass spectrometry analysis in urine and plasma. The role of vascular smooth muscle cells from human aorta (HA-VSMCs) was also investigated by analyzing the intra and extracellular metabolites in response to a pro-atherosclerotic stimulus. Statistically significant variation was considered if *p* value < 0.05 (Mann-Whitney test). Urinary trimethylamine N-oxide (TMAO), arabitol and spermidine showed higher levels in the CVrisk group compared with a control group; while glutamine and pantothenate showed lower levels. The same trend was found for plasma TMAO and glutamine. Plasma choline, acetylcholine and valine were also decreased in CVrisk group, while pyruvate was found increased. In the secretome of HA-VSMCs, TMAO, pantothenate, glycerophosphocholine, glutathion, spermidine and acetylcholine increased after pro-atherosclerotic stimulus, while secreted glutamine decreased. At intracellular level, TMAO, pantothenate and glycerophosphocholine increased with stimulation. Observed metabolic deregulations pointed to an inflammatory response together with a deregulation of oxidative stress counteraction.

## 1. Introduction

Cardiovascular disease continues being the leading cause of premature death worldwide despite continuing efforts in primary prevention. The predominant pathology of cardiovascular disease is atherosclerosis, an immunoinflammatory disease of arterial vessels. Formation of the atheroma plaque and progressive arterial obstruction takes place silently and asymptomatically, making it extremely challenging to promptly identify those individuals at high cardiovascular risk to prevent a fatal event. Main risk factors are age, male gender, lipid profile, blood pressure, smoking habits and diabetes. Despite that over 50% of the observed decrease in mortality caused by coronary heart disease can be attributed to changes in those risk factors, further knowledge is needed to better stratify individual risk and improve prevention [1,2]. No early diagnostic markers are available to date that may undoubtedly predict future events on healthy subjects or stratify individual risk. In this clinical scenario, there is an urgent need to find out novel molecular indicators, alone or combined with existing ones, together with a better knowledge of the operating mechanisms in atherosclerosis development.

The metabolome reflects the ultimate response of an organism to a patho(physio)logical condition and provides an integrated profile of biological status and metabolic health. Metabolomics integrates the comprehensive analysis of low molecular weight molecules in biological systems, providing a global picture of individual response to intrinsic and extrinsic exposures to genetic, dietary, lifestyle and gut microbial factors [3]. Accordingly, defining the chemical phenotypes of health or disease using metabolic signatures is gaining attraction in cardiovascular risk stratification [4,5,6,7]. In this line, an improved prediction of cardiovascular events based on metabolic features mainly composed by intermediates of fatty acid oxidation has been shown [8]. Metabolic signatures associated with coronary artery disease plaque phenotypes [4] and correlating with severity of coronary stenosis [9] have been recently described. Previous studies from our group have evidenced metabolic patterns associated with increased cardiovascular risk in various clinical contexts. In particular, urinary metabolic alterations were identified in hypertensive subjects with increased cardiovascular risk (i.e., resistant hypertension or albuminuria development) [10,11]. Specific urinary panels were also shown to be altered during atherosclerosis development in a high fat diet animal model and in human subjects in response to an acute coronary syndrome [12]. In a different approach, the study of molecular mechanisms taking place directly in the atherosclerotic aorta revealed novel molecular actors pointing to oxidative stress and arterial remodeling [13]. In a step further in cardiovascular prevention, the aim of this study is to evaluate the potential of the previously identified metabolic signatures in association with cardiovascular risk in a cohort of subjects with programmed coronary arterial bypass grafting (CABG). Besides, a potential role for the vascular smooth muscle cells from human aorta (HA-VSMCs) in observed changes was also investigated.

## 2. Materials and Methods

### 2.1. Patient Selection and Samples Collection

A total of 27 patients undergoing CABG at Fundación Jiménez Díaz Hospital (Madrid, Spain) were recruited (CVrisk group). Inclusion criteria were diagnosis of coronary artery disease, and programmed CABG surgery. Exclusion criteria were previous open cardiac surgery, valvulopathy, neoplasia, emergency, hemodynamic instability, treatment with corticoids or immunosuppressants, active infection or in-hospital exitus. As control group (C), a cohort of 24 healthy subjects was recruited from Donation Unit at the same hospital without previous history of hypertension, diabetes or cardiovascular disease. Subjects’ clinical characteristics are compiled in Table 1. A spot urine sample was collected from each participant in a sterile container. Blood samples were collected in EDTA tubes. Urine and blood samples were centrifuged and supernatants were collected and stored at −80 °C until analysis. For surgery patients, samples were collected at two timepoints: before surgery (CVrisk group) and at clinical follow-up after 3 months (recovery, R).

### 2.2. In Vitro Human Cell Cultures

Primary cultures of vascular smooth muscle cells were obtained from a whole aortic arterial segment of patient undergoing cardiac surgery (HA-VSMC) according to previously described [14]. The arterial segment was cleaned and cut into small pieces. They were then incubated with Dulbecco’s modified Eagle’s medium (DMEM-F12; Lonza Walkersville) containing 0.1% collagenase type 1 (Gibco) overnight at 37 °C. After incubation, the reaction was stopped with 0.5% fetal bovine serum (FBS) in DMEM-F12 and after centrifugation at 1200 rpm for 5 min, the explants were seeded together with the cells in DMEM-F12 medium supplemented with 0.1 mg/mL heparin (Merck Life Science), 30 μg/mL endothelial cell growth factor (ECGF; Merck Life Science), 1% penicillin/streptomycin (P/E; Lonza Walkersville), 1% L-glutamine (Lonza Walkersville), 10% Fungizone (Lonza Walkersville) and 20% FBS. Petri dishes used for seeding were pre-coated with 0.5% sterile gelatin. After 3–5 days of incubation, HA-VSMCs were negatively selected with the human CD31 antibody (BDbiosciences) and secondary antibody-associated magnetic beads (Dynabeads^®^ anti-mouse IgG from the CELLection™ Pan Mouse IgGy kit; Invitrogen). The cell populations were then separated, the endothelial aortic cells were retained on the surface of the tube in contact with the magnet, while the HA-VSMCs remained in suspension without adhering to the magnet. HA-VSMCs were seeded and maintained in DMEM medium (Lonza Walkersville) with 10% FBS.

### 2.3. Stimulation Assay

HA-VSMCs were seeded and grown in four 60-mm polystyrene plates (Corning, Cultek), indicated for the cultivation of adherent cells until monolayer formation, and were allowed to reach confluence. Once FBS depletion was completed for 18 h at 0.5%, stimulation was performed as follow: DMEM medium in control plate vs DMEM medium with an inflammatory cocktail of Interleukin-1 (10 ng/mL), Interferon-α (10 ng/mL), Angiotensin-II (1 ng/mL) in stimulated plates. After 1 h, 4 h and 24 h of contact the medium was collected (secretome), and the cells were washed five times with cold PBS, lifted from the surface of the plates using a scraper and processed for metabolites extraction in methanol. The experiment was performed four times (technical replicates).

### 2.4. Metabolites Extraction

For metabolite analysis, protein removal in urine and secretome from HA-VSMCs was performed by precipitation in acetonitrile/0.1% formic acid (1:1) based on previous analyses [10,11,12]. In the case of plasma samples, protein removal was performed with pre-chilled methanol (1:1). Metabolites were extracted from HA-VSMCs in 50% methanol [13]. In all cases, following centrifugation, the supernatant was diluted with mobile phase A (0.1% formic acid in Milli-Q water) and filtered through 0.22 μm for subsequent LC-MS/MS analysis.

### 2.5. Targeted Analysis by Liquid Chromatography and Mass Spectrometry

Targeted mass spectrometry analysis was accomplished by Selected Reaction Monitoring (SRM-LC-MS/MS) methodology as previously published [12,15]. A 6460 Triple Quadrupole QQQ on-line connected to HPLC (1200 Series) controlled by Mass Hunter Acquisition Software (v4.01) (Agilent Technologies) was used. Samples were analyzed in a reversed-phase column (Atlantis T3, 3 μm, 2.1 × 100 mm, Waters) thermostatically controlled at 40 °C. A sample volume of 10 μL was injected and separation took place at 0.4 mL/min in an acetonitrile gradient. Instrumental conditions (collision energy and fragmentor potential) were optimized for each SRM transition (see Supplementary Table S1). Delta electron multiplier voltage was set to 600 V in negative ion mode or 400 V in positive ion mode. Peak areas were calculated from individual signals for intergroup comparison. Urine metabolic content was normalized by creatinine values. A detailed list of the measured metabolites is compiled in Supplementary Table S2.

### 2.6. Statistical Analysis

The ROUT method was applied to detect outliers based on the FDR, setting Q value to 5%. Mann-Whitney non-parametric test (95% confidence level) was performed with GraphPad Prism 6 software (version 6.01). Individual and combined diagnostic capacity was evaluated by receiving operating characteristics (ROC) curves using Metabonalyst 5.0 platform. The combined multivariate ROC curves were obtained using random forest algorithm as built-in method. The area under the curve (AUC) and the 95% confidence interval were estimated for both individual and multivariate analysis.

## 3. Results

A cohort of subjects with programmed CABG was recruited as a representative population of high cardiovascular risk (CVrisk group) (Table 1). The control group (age 48 ± 6; 38% male) have no history of diabetes, hypertension or cardiovascular disease. Those metabolites previously identified by our group with significant alteration in urine and aorta from atherosclerotic rabbits [12,13], in urine from hypertensive subjects with higher cardiovascular risk in terms of albuminuria development [10], and in urine from subjects at the onset of an acute coronary syndrome [12] were here analyzed by targeted mass spectrometry to investigate their alteration during human atherosclerosis development. Supplementary Table S2 compiles these altered metabolites previously identified in the referred studies and here analyzed in a new cohort and in four different matrices.

**Figure 1:**
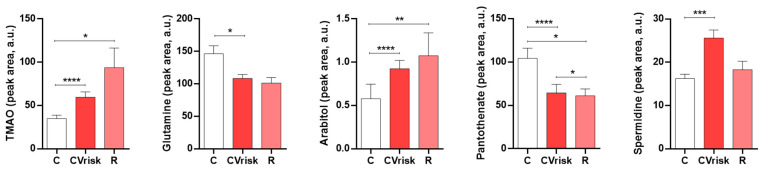
Metabolite abundance in urine in subjects with programmed bypass surgery (CVrisk group) compared to healthy subjects (C group). Data during clinical follow-up Table 0. * *p* value < 0.05, ** *p* value < 0.01, *** *p* value < 0.001, **** *p* value < 0.0001.

We first analyzed the metabolites abundance in urine according to previous findings, confirming their association with cardiovascular risk for five metabolites: trimethylamine N-oxide (TMAO), arabitol and spermidine showed higher levels in pre-CABG patients compared with the control group; while glutamine and pantothenate showed lower levels (Figure 1). When analyzed 3 months after CABG surgery, spermidine levels normalize towards control values, contrary to TMAO and arabitol which showed increased alteration.

**Figure 2:**
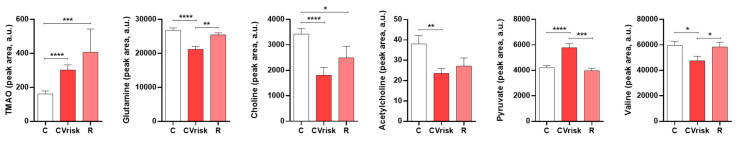
Metabolite abundance in plasma in subjects with programmed bypass surgery (CVrisk group) compared to healthy subjects (C group). Data during clinical follow-up 3 months after Scheme 0. * *p* value < 0.05, ** *p* value < 0.01, *** *p* value < 0.001, **** *p* value < 0.0001.

Metabolites were also analyzed in plasma (Figure 2). The same variation trends previously observed in urine were found in plasma for TMAO and glutamine, which normalized to control values following surgery. Choline, acetylcholine and valine were found to be decreased in plasma from CVrisk subjects, while pyruvate was found to be increased. Normalized levels were observed post-surgery also for valine and pyruvate. 

The metabolites diagnostic potential was evaluated by ROC curves in terms of sensitivity and specificity. Table 2 shows the area under the curve (AUC) obtained for each metabolite found significantly altered in urine or plasma and for combined models.

Best performing biomarkers of cardiovascular risk were arabitol and TMAO in urine, and glutamine, TMAO and choline in plasma (AUC = 0.720–0.763). All urine metabolites resulted in an AUC = 0.889 when combined. Combined plasma metabolites showed AUC = 0.902. Glutamine and TMAO were found significantly altered in both matrices giving AUC = 0.743 and AUC = 0.779, respectively, if analyzed in both biological fluids.

**Figure 3:**
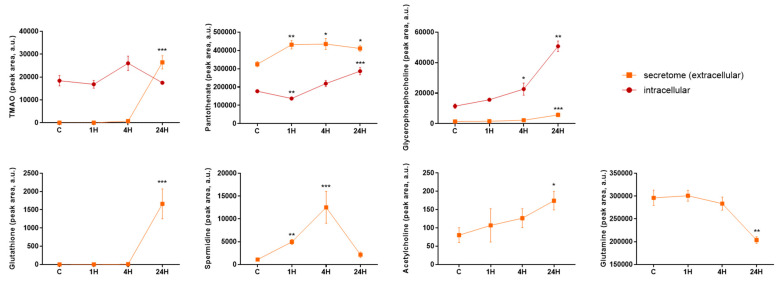
Intracellular and extracellular metabolic content of HA-VSMCs exposed to pro-atherogenic stimulus. Metabolites were extracted from HA-VSMCs and from the secreted media after 1 h, 4 h and 24 h following stimulation. Statistical significance was calculated for every time point versus control group (C). * *p* value < 0.05, ** *p* value < 0.01, *** *p* value < 0.001.

Finally, the potential contribution of HA-VSMCs to metabolic de-regulation observed in biological fluids was investigated. HA-VSMCs intracellular and extracellular (secretome) metabolic content was analyzed in response to a pro-atherosclerotic stimulus at different time points (1 h, 4 h and 24 h) (Figure 3). Increased secreted values compared to cells without stimulation (control) were found for TMAO, pantothenate, glycerophosphocholine, glutathion, spermidine and acetylcholine. Secreted glutamine was found to be decreased in the pro-atherosclerotic environment. Pantothenate and spermidine showed the earliest response, while extracellular levels of glutathione, glutamine, acetylcholine and glycerophosphocholine significantly varied only after 24 h. At an intracellular level, TMAO, pantothenate and glycerophosphocholine varied with stimulation.

## 4. Discussion

Metabolomics is an approach that has shown great potential in cardiovascular research, opening new avenues for the study of underlying mechanisms and revealing new therapeutic targets and markers. In this work, we have applied targeted metabolomics to four different matrices, urine, plasma, HA-VSMCs and secretome. That allows us, on the one hand, to better understand the metabolisms associated with the pathology and, on the other hand, to identify the most robust molecules as markers. Our study reveals an imbalance in the defense mechanisms against oxidative stress and a response to atherosclerosis related inflammation. Additionally, it identifies TMAO and glutamine as the most altered molecules in the pathology in different accessible fluids.

### 4.1. Insufficient Oxidative Stress Counteraction during Atherosclerosis Development

Oxidative stress is one of the main operating mechanisms in atherosclerosis [16], induced by inflammation, mitochondria, autophagy and apoptotic processes taking place in the course of the disease [17]. Elevated levels of reactive oxygen species (ROS) resulting in antioxidative stress acts as a crucial mechanical effect in atherosclerosis progression. In this line, increasing antioxidant capacity may be one of the key points in future atherosclerosis therapy.

In response to the exposure of ROS released in oxidative stress conditions, synthesis of polyamines is induced. Spermidine is a natural polyamine with protective effects in human cardiovascular health [18]. In particular, spermidine is exported from the cell by the polyamine transporter TPO1 in response to oxidative stress [19]. In agreement with an attempt to counteract oxidative stress conditions, we observed here increased spermidine in the VSMCs secretome following inflammatory stimulus, and in urine of subjects with programmed CABG surgery, following normalization to pre-surgery values 3 months after CABG intervention. We previously found spermidine levels increased in urine collected after an acute coronary syndrome [12]. Endothelial nitric oxide synthase (eNOS) has a protective function in the cardiovascular system by generating antioxidant nitric oxide (NO). Arginase competes with eNOS for arginine and converts it in ornithine, a precursor of spermidine. Valine inhibits arginase, and reduced levels here observed for valine may also reflect a deleterious balancing in oxidative stress counteraction and increased production of spermidine. 

Glutamine is another metabolite which counteracts oxidative stress and exerts an anti-inflammatory role, showing a negative correlation between its circulating levels and cardiometabolic disease [20]. As a precursor of glutathione, glutamine plays a key role in the prevention of oxidative damage and contributes to the maintenance of the endothelial function of the blood vessels [21]. Glutamine was here found decreased in both biological fluids from cardiovascular risk patients, and also in the secretome of VSMCs pointing to a deleterious protective effect against atherosclerosis development. These data are in consonance with our previously reported diminished levels in urine from middle age (50–70 years) and elderly (>70 years) subjects with cardiovascular risk factors [22].

### 4.2. Metabolic Response to Inflammation in Atherosclerosis Development

Choline and acetylcholine were also found decreased in plasma of CABG subjects. Choline is a structural component of cell membranes; it participates in the transport and metabolism of cholesterol and helps maintaining circulating homocysteine levels. Increased plasma homocysteine is an independent risk factor for atherosclerosis development which can be caused by decreased methylation of homocysteine to form methionine, being choline the methyl donor [22]. Our data support a role for choline in the increased cardiovascular risk associated with circulating homocysteine. Choline is a precursor of acetylcholine and the involvement of its receptor a7 nicotinic acetylcholine receptor (α7nAChR) in the development of atherosclerosis is an expanding field. In vivo studies revealed both anti- or pro-atherogenic effects of α7nAChR stimulation [23]. In this study, the observed reduced levels of plasma acetylcholine are in consonance with the same trend observed for its precursor choline. On the contrary, VSMCs secreted higher amount of acetylcholine after inflammatory stimulation, in line with inflammation and oxidative stress suppression observed in mice aorta after vascular injury mediated by α7nAChRs activation [24].

Choline is also one of the trimethylamine-containing nutrients and, as such, a TMAO precursor. Plasma TMAO levels depend on liver production, ingestion and renal excretion. Exposure of VSMCs to TMAO induce inflammation and suggest atherosclerosis enhancement by a TMAO-dependent mechanism [25]. In our study, secreted TMAO levels increase in response to a pro-inflammatory stimulus in what can be seen as a contribution to observed higher levels in plasma and urine from cardiovascular risk subjects with moderately preserved renal function. Urinary TMAO was found increased in young (30–50 years), middle age (50–70 years) and elderly (>70 years) population with cardiovascular risk factors in our previous study [22], in agreement with data shown here and pointing to a strong association with CV risk independently of age. 

**Figure 4:**
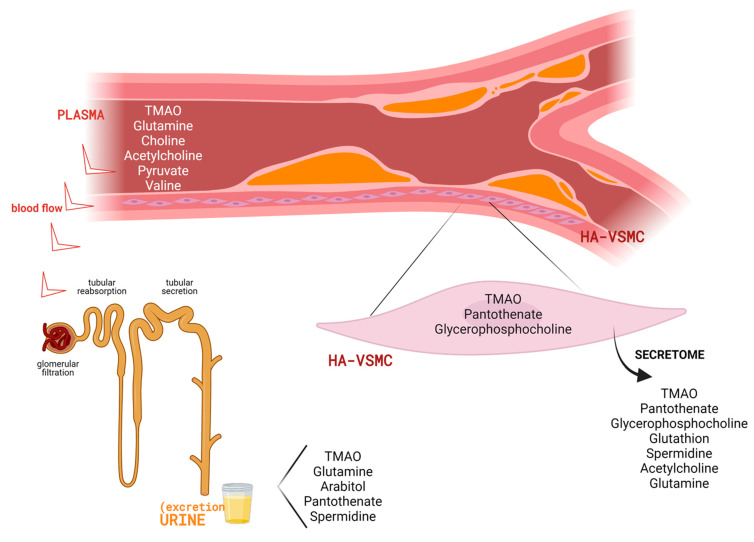
Overview of metabolic alterations identified in association with CVrisk and atherosclerosis development. Specifically, changes were observed in urine and plasma from subjects with programmed CABG surgery, and in the intracellullar and extracellular (secretome) content of human aortic VSMCs (HA-VSMCs) after exposure to a pro-atherosclerotic stimulus. Created with BioRender.com (accessed on 29 July 2021).

Figure 4 shows a representative image including all metabolites with significantly altered levels in the different matrices investigated. It has to be considered that not all metabolites could be detected in all matrices (urine, plasma, HA-VSCMs and cellular secretome). Discussion was thus focused on confirmed significant differences between CVrisk subjects and stimulated HA-VSMCs in comparison with their respective controls.

### 4.3. Urine and Plasma Metabolites with Diagnostic Potential in Cardiovascular Risk Evaluation

Metabolites with diagnostic potential should be easily accessible in routine analyses. In this sense, urine and plasma are the most used biological fluids in daily clinical practice. Regarding cardiovascular risk estimation, novel tools based on molecular profiling should ideally respond to subjacent atherosclerosis development despite its silent progression. In this study, we show the stronger potential of multimarker panels compared to metabolites individual performance. This combination may include best performing metabolites according to sensitivity/specificity criteria, or analyses in various biological matrices.

We and others have previously demonstrated urinary metabolites alteration after an acute coronary syndrome (ACS). In that case, two metabolic responses may be overlapping, i.e., responding both to the acute event itself and to the pathophysiological deregulation taking place in the atherosclerotic arterial vessel. Arabitol and spermidine show here the same increased trend than previously observed in ACS. In the case of arabitol, no recovery towards control values was observed in any case (following the acute event, or after 3 months from CABG surgery). However, spermidine reflects recovery following surgery indicating a quicker response to therapeutic intervention.

TMAO and glutamine showed the same trend in urine and plasma from CABG patients. In the aorta from atherosclerotic rabbits, the same trend was observed for glutamine suggesting a direct reflection in fluids of diminished content in the atherosclerotic vessel. However, TMAO showed reduced aortic levels pointing to a plausible diet effect on its urine/plasma levels. Plasma levels of choline, acetylcholine, pyruvate and valine are also in agreement with aortic levels during atherosclerosis progression.

The analysis of combined and individual features and their predictor potential showed, as expected, that metabolic panels present a better performance than individual molecules in risk prediction. Particularly, in this study, the combination of five urine metabolites or six plasma metabolites led to marker panels with an AUC of 0.9 while individual features average AUC was 0.7. Previous analyses performed in plasma in large cohorts have showed the value of metabolomics for biomarker discovery. Phenyalanine, MUFA relative to total fatty acids (MUFA%), Omega-6 and DHA combined are described to improve prediction of cardiovascular risk in comparison with established risk factors [26]. More recently, a study performed in more than 10,500 individuals showed that five phosphocholines have a comparable discrimination of risk of coronary heart disease with classic estimators [27]. Regarding urine and CV risk estimation, large studies are missed. By analysing the same set of metabolites in plasma, urine and HA-VSMCs here, we cannot only estimate the predicting value of the features but also the different performance of the measurable matrix. Our results demonstrate the value of urine marker panels, in accordance to our previous studies, and showed a similar performance to those performed in plasma. Interestingly, in this particular case, there is not an improvement when combining plasma and urine measurements. Pending studies in large and multiple cohorts, these metabolites being quantifiable in accessible fluids may aid in assessing individual CV risk during asymptomatic stages in atherosclerosis development, facilitating earlier diagnosis and patients’ risk stratification. 

## 5. Conclusions

Here we demonstrate how molecular deregulation directly observed in the atherosclerotic aortic tissue can be detected in an accessible biological fluid, setting the basis for novel diagnostic tools complementing existing algorithms in cardiovascular risk prediction or individual stratification. Previous metabolic alterations shown in response to an acute coronary syndrome can also take place during atherosclerosis development. We show here that metabolite deregulations identified during atherosclerosis development point to an inflammatory response together with a defective or insufficient action of mechanisms counteracting oxidative stress.

## Figures and Tables

**Table 1:** Clinical data of CABG patients included in the study.

	CVrisk
Age	68 ± 9
Sex, male (%)	52
Diabetes mellitus (%)	26
Arterial hypertension (%)	89
Smoking habits (%)	26
Estimated glomerular filtration rate (mL/min/1.73 m^2^)	66 ± 21

**Table 2:** Metabolites potential as biomarkers of cardiovascular risk, evaluated by ROC curves calculation. AUC: area under the curve. CI: confidence interval.

	AUC	CI (95%)
Individual metabolites urine		
Arabitol	0.763	(0.671–0.860)
Glutamine	0.528	(0.419–0.639)
Pantothenate	0.686	(0.590–0.800)
Spermidine	0.679	(0.573–0.785)
TMAO	0.720	(0.612–0.816)
Individual metabolites plasma		
Acetylcholine	0.682	(0.575–0.772)
Choline	0.740	(0.635–0.829)
Glutamine	0.748	(0.656–0.836)
Pyruvate	0.723	(0.627–0.811)
TMAO	0.741	(0.641–0.832)
Valine	0.617	(0.488–0.724)
Combined metabolites		
Urine	0.889	(0.778–0.980)
Plasma	0.902	(0.823–0.983)
Glutamine (urine and plasma)	0.743	(0.602–0.855)
TMAO (urine and plasma)	0.779	(0.656–0.909)

## Data Availability

Data are contained within the article or in Supplementary Material.

## Supplementary Materials

The following are available online at https://www.mdpi.com/article/10.3390/antiox10091369/s1, Table S1: Experimental conditions for metabolites analysis by SRM, Table S2: Metabolites previously identified by our group in association with cardiorenal risk uEA: early atherosclerosis, detected in urine [12]. aEA: early atherosclerosis, detected in aorta [13]. ACS: acute coronary syndrome at the onset [12]. ALB: subjects with moderately increased albuminuria [10].
