# Supplementary material for: Metabolic Alterations Identified in Urine, Plasma and Aortic Smooth Muscle Cells Reflect Cardiovascular Risk in Patients with Programmed Coronary Artery Bypass Grafting
Source: Antioxidants (Basel). 2021 Aug 27;10(9):1369. doi: 10.3390/antiox10091369 (PMC8466954; doi:10.3390/antiox10091369)
Supplement: Supplementary file 1 [file antioxidants-10-01369-s001.zip › antioxidants-1347787-supplementary.pdf]

Table S1. Instrumental conditions for target analysis of metabolites by (SRM)-LC-MS/MS.

Metabolite name	Precursor mass (m/z)	Fragment mass (m/z)	Dwell time (ms)	Fragmentor (V)	Collision energy (V)	Polarity
1-methylhydantoin	113.1	69.1	50	90	10	Negative
2-hydroxyphenylacetic acid	151.2	107.1	50	72	10	Negative
3-hydroxybutyric acid	103.1	59.1	50	60	10	Negative
3-ureidopropionic acid	133.13	115.1	20	70	10	Positive
acethylcholine	147.2	88.1	20	60	14	Positive
alanine	90.1	44.1	40	130	22	Positive
arabitol	153.2	61.2	20	60	34	Positive
aspartic acid	134.11	74.2	20	60	10	Positive
choline	105.18	64.2	20	60	10	Positive
cyclohexanol	101.2	84	20	130	10	Positive
glucuronic acid	193.1	112.9	50	120	10	Negative
glutamic acid	147.9	102	40	175	18	Positive
glutamine	147.15	84.1	20	84	18	Positive
glutathione	308	84.1	20	175	40	Positive
glyceric acid	107.8	61.2	20	144	10	Positive
glycerophosphocholine	258.2	104	20	132	10	Positive
guanidoacetic acid	118.1	76.1	20	120	10	Positive
malic acid	133	115.1	50	84	10	Negative
N-acetylalanine	132.14	44.3	40	120	26	Positive
N-acetylneuraminic acid	308.3	87	50	90	10	Negative
oxalacetic acid	131.1	86.8	50	100	10	Negative
pantothenic acid	220.4	90.1	20	80	14	Positive
p-benzoquinone	109.9	69	20	74	10	Positive
phosphocholine	185.1	57.2	20	60	14	Positive
putrescine	89.2	72.2	20	60	10	Positive
pyruvic acid	87.1	43.3	50	60	10	Negative
scyllo-inositol	181.2	137.1	20	130	14	Positive
spermidine	146.3	72.2	20	60	14	Positive
threitol	121.13	75.1	20	60	10	Negative
threonine	120.13	102.9	20	130	18	Positive
TMAO	76.1	59	20	175	22	Positive
tyramine	138.2	121.1	20	60	10	Positive
valine	118.16	58.1	20	130	30	Positive

Table S2. Metabolites previously identified by our group in association with cardiorenal risk uEA: early atherosclerosis, detected in urine [12]. aEA: early atherosclerosis, detected in aorta [13]. ACS: acute coronary syndrome at the onset [12]. ALB: subjects with moderately increased albuminuria [10].

Metabolite name	Previous studies	Reference
1-methylhydantoin	ACS	[12]
2-hydroxyphenylacetic acid	ACS	[12]
3-hydroxybutyric acid	ACS, ALB	[12], [10]
3-ureidopropionic acid	ALB	[10]
acetylcholine	aEA	[13]
alanine	ACS	[12]
arabitol	ACS	[12]
aspartic acid	aEA	[13]
choline	aEA	[13]
cyclohexanol	ACS	[12]
glucuronic acid	aEA	[13]
glutamic acid	aEA, ALB	[13], [10]
glutamine	aEA	[13]
glutathione	aEA	[13]
glyceric acid	ALB	[10]
glycerophosphocholine	aEA	[13]
guanidoacetic acid	aEA, ALB	[13], [10]
malic acid	ALB	[10]
N-acetylalanine	aEA	[13]
N-acetylneuraminic acid	ACS	[12]
oxalacetic acid	ALB	[10]

pantothenic acid	ALB	[10]
p-benzoquinone	ACS	[12]
phosphocholine	aEA	[13]
putrescine	ACS	[12]
pyruvic acid	aEA, ALB	[13], [10]
scyllo-inositol	ACS	[12]
spermidine	ACS	[12]
threitol	aEA	[13]
threonine	aEA	[13]
TMAO	aEA	[13]
tyramine	aEA	[13]
valine	aEA	[13]
